# A Review of Watershed Implementations for Segmentation of Volumetric Images

**DOI:** 10.3390/jimaging8050127

**Published:** 2022-04-26

**Authors:** Anton Kornilov, Ilia Safonov, Ivan Yakimchuk

**Affiliations:** 1 Schlumberger Moscow Research, Leningradskoe Highway, 16a, 125171 Moscow, Russia; akornilov@slb.com (A.K.); iyakimchuk@slb.com (I.Y.); 2 Computer Science and Control Systems Department, National Research Nuclear University MEPhI, Kashirskoye Highway, 31, 115409 Moscow, Russia

**Keywords:** segmentation, watershed algorithm, waterline, flooding, Euclidean distance transform, benchmarking, performance, memory consumption

## Abstract

Watershed is a widely used image segmentation algorithm. Most researchers understand just an idea of this method: a grayscale image is considered as topographic relief, which is flooded from initial basins. However, frequently they are not aware of the options of the algorithm and the peculiarities of its realizations. There are many watershed implementations in software packages and products. Even if these packages are based on the identical algorithm–watershed, by flooding their outcomes, processing speed, and consumed memory, vary greatly. In particular, the difference among various implementations is noticeable for huge volumetric images; for instance, tomographic 3D images, for which low performance and high memory requirements of watershed might be bottlenecks. In our review, we discuss the peculiarities of algorithms with and without waterline generation, the impact of connectivity type and relief quantization level on the result, approaches for parallelization, as well as other method options. We present detailed benchmarking of seven open-source and three commercial software implementations of marker-controlled watershed for semantic or instance segmentation. We compare those software packages for one synthetic and two natural volumetric images. The aim of the review is to provide information and advice for practitioners to select the appropriate version of watershed for their problem solving. In addition, we forecast future directions of software development for 3D image segmentation by watershed.

## 1. Introduction

Image segmentation by the watershed algorithm, because of its innate ability to produce closed-regions, has many applications in science, medicine, and industry. Despite the great advances of deep neural networks (DNN) intended for segmentation, watershed remains an important technique for solving some specific segmentation problems. One of the typical uses for watershed is separation of touching or overlapping objects in a binary image to employ an instance segmentation, when semantic segmentation has been previously performed by another technique. Currently, DNN and watershed are often used jointly [1,2,3].

Many publications mention the use of watershed. We enumerate here only a few of them to demonstrate the huge variety of practical applications: a pore network extraction from 3D X-ray computed tomography (CT) images of porous media [4]; a characterization of ceramic proppant to the crush resistance by comparison of particles from two CT images [5]; a segmentation of melanin granules in the retinal pigment epithelium for images of optical coherence tomography [6]; karyotyping of chromosome images obtained by means of an optical microscope [7]; a location of spruces in a young stand with an unmanned aerial vehicle [8]; and cell segmentation and detection in live-cell fluorescence microscope imaging [9].

Typically, training courses and guides on computer vision or image processing only explain the general concept of a watershed. The name refers metaphorically to a geographical watershed, which separates adjacent drainage basins. A 2D image or some of its derivatives are treated as topographic relief (landscape). In classical watershed, local minima in the relief are initial basins. In a marker-controlled method, the markers are initial basins. Starting from the minimum of lowest height, the water gradually fills up all catchment basins. Image elements where water from different basins meets are called by watershed lines (WL) or dams. The process ends when the water reaches the maximum peak of the relief, and as a result, every catchment basin (i.e., segment) gets covered by WL. There is another explanation. Image elements at which a drop of water falls to the given local minimum form a catchment basin. Image elements at which a drop of water can fall to different basins form ridges or WL. Even if the watershed description was completed via a set theory of mathematical morphology (for example, see the well-known book by Gonzalez and Woods [10]), it does not reflect the peculiarities of the algorithms and their implementations in software.

**Figure 1:**
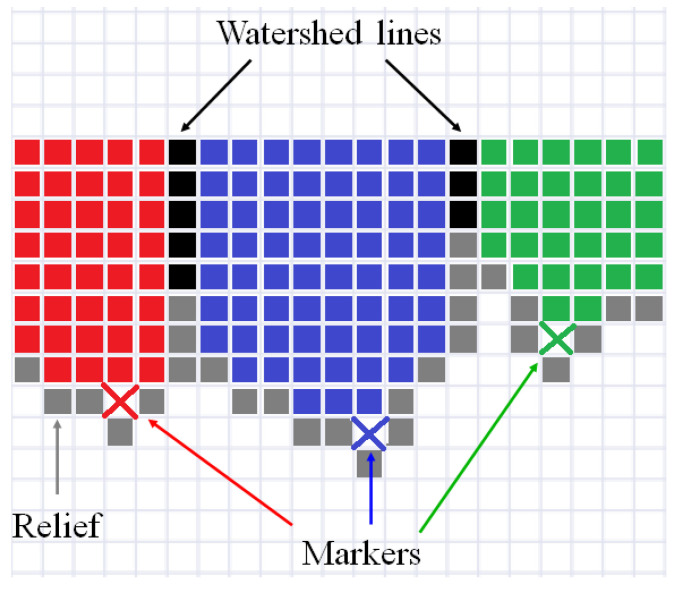
Illustration of marker-controlled watershed for one-dimensional signal.

Figure 1 illustrates the simplest explanation of the watershed idea for an one-dimensional signal. Pixels of relief are in a gray. Initial markers are designated by the crosses in red, blue, and green. Filling starts from the initial markers, and as result, three segments (in red, blue, and green, respectively) are formed. Watershed lines (in black) are placed between the segments.

There are many scientific studies that apply ready-to-use implementations of watershed from existing open-source or commercial software. For instance, the papers [11,12] mention the watershed algorithm from the Insight Segmentation and Registration Toolkit (ITK) library for brain extraction from magnetic resonance images and analysis of glass foams using X-ray micro-CT (μCT); the papers [13,14] refer to the algorithm from the Mahotas library for a deep structured learning method for neuron segmentation from 3D electron microscopy and tracking of surface-labeled cells in time-lapse image sets of living tissues; the papers [15,16] mention the algorithm from the Scikit-image (Skimage) library for pore network extraction from micro-CT data; the publication [17] describes segmentation of blood cells by MATLAB^®^. Meanwhile, [18] refers to watershed segmentation of micro-CT images of rock samples by Avizo^®^. Frequently, researchers use functions from software without a deep understanding of the algorithms’ details and limitations. Sometimes, they have no information about alternative solutions. This can lead to investigations with disappointing results.

In 2018 we published a benchmarking study of marker-based watershed implementations in open-source software libraries called from the Python programming language [19]. Recently, we received much positive feedback and many requests for an update of this evaluation. Our previous review was mainly focused on processing of 2D images, we only briefly handled a single 3D synthetic sample. In this paper, we consider processing of volumetric images by means of marker-based watershed implementations in open-source and commercial software without any restriction on programming language. A description of the watershed algorithm options is extended in comparison with our previous review. In addition, the paper contains a performance comparison of 3D Euclidean distance transform (EDT) implementations in the software under investigation, because EDT and watershed are often applied one-by-one in a processing pipeline.

Our current review pursues three main goals: to reveal in a simple manner the peculiarities and options of watershed algorithms; to compare various software implementations of watershed for image segmentation; and to forecast future directions of software development for 3D image segmentation by watershed. We hope our paper will be useful for practitioners who use watershed in scientific or industrial applications as well as for corresponding software developers to improve their products.

This paper is organized as follows: Section 2 discusses watershed algorithms and factors that affect the results as well as the resources required; Section 3 describes seven open-source libraries and three commercial products included in benchmarking. Section 4 demonstrates, on a simple 2D example, the differences in outcomes of several implementations and presents the measured execution time and amount of consumed memory during processing of one synthetic and two natural volumetric images. Section 5 contains the discussion about the current state of affairs and future advances in image segmentation via watershed.

## 2. Description of Watershed Algorithms Applied in Software

Because the considered algorithms are intended for processing both 2D and 3D images (strictly speaking, n-dimensional images can be processed), we use the term “image element” together with the terms pixel and voxel. Beucher and Lantuéjoul [20] introduced watershed for segmentation of grayscale images, although the watershed transformation as an operation of mathematical morphology was described a few years earlier [21,22]. To solve the oversegmentation problem caused by a huge number of initial basins started from each local minima of an image marker-controlled (or seeded) watershed was proposed [23]. Vincent and Soille [24] generalized watershed for an n-dimensional image and depicted an algorithm based on an immersion process analogy, in which the flooding of relief by water is simulated using a queue (first-in-first-out (FIFO) data structure) of image elements.

Beucher and Meyer [25] developed effective algorithms, in which a flooding process is simulated by using a priority queue [26], where a priority is a value of a relief element, and a lower value corresponds to a higher priority. An illustrative example of a pseudocode describing steps of marker-controlled watershed by Beucher and Meyer is reported in Algorithm 1. Here, we explain the variables and steps of this algorithm:

BM1 Image (relief) elements $(i,r_{i})\in R$ corresponding to markers (labels) $(i,m_{i})\in M$ that have at least one unmarked neighbor $\left(j,m_{j}\right)\in N_{D}\left(i,m_{i}\right)$ (i.e., marker of background bg) are added to the priority queue PQ; see lines 4–9 in Algorithm 1.
BM2 Element with the highest priority is extracted from the queue; if the priority queue is empty, then the algorithm terminates; see lines 10–11.
BM3 Marker of the extracted element propagates on its unmarked neighbors; see lines 12–13.
BM4 The neighbors marked in the previous step are inserted into the priority queue with the same priority or lower than the extracted element (if neighbor has higher relief value); then, go to step BM2; see line 14.

**Algorithm 1** The marker-controlled watershed by Beucher and Meyer [25].**Require:** 
|R|=|M|1:**function**Watershed(R,M)            ▹ a relief and a markers as parameters2:    PQ←∅                          ▹ the priority queue3:    bg←0                          ▹ value of background4:    **for** $\left(i,r_{i}\right)\in R\wedge \left(i,m_{i}\right)\in M\wedge m_{i}\neq bg$ **do**5:        **for** $\left(j,m_{j}\right)\in N_{D}\left(i,m_{i}\right)\wedge m_{j}=bg$**do** ▹ iterate over neighbors of element $\left(i,m_{i}\right)$6:           $push(r_{i},i,PQ)$             ▹ push *i* into PQ with priority level $r_{i}$7:           **break**8:        **end for**9:    **end for**10:    **while** |PQ|≠0 **do**11:        i←pop(PQ)       ▹ pop an element coordinate from the priority queue12:        **for** $\left(j,m_{j}\right)\in N_{D}\left(i,m_{i}\right)\wedge m_{j}=bg$ **do**13:           $M\leftarrow \left(M\setminus \left(j,m_{j}\right)\right)\cup \left(j,m_{i}\right)$              ▹ mark an element14:           $push(max\left(r_{i},r_{j}\right),j,PQ)$15:        **end for**16:    **end while**17:    **return** *M*18:**end function**

One can see that the algorithm by Beucher and Meyer does not form WL. Frequently, watershed lines are valuable segmentation outputs. Meyer [27] described the algorithm with WL construction. Pseudocode of this method is presented in Algorithm 2. Appendix A contains source codes of Algorithms 1 and 2 for processing 3D images with 26-connectivity in the Python programming language.
**Algorithm 2** The marker-controlled watershed with WL construction by Meyer [27].**Require:** 
|R|=|M|1:**function**WatershedWL(R,M)2:    PQ←∅3:    bg←04:    $wl\leftarrow max(\left\{m_{i}|\left(i,m_{i}\right)\in M\right\})+1$              ▹ value of WL marker5:    $V\leftarrow \{i|\left(i,m_{i}\right)\in M\wedge m_{i}\neq bg\}$                 ▹ visited elements6:    **for** $\left(i,r_{i}\right)\in R\wedge \left(i,m_{i}\right)\in M\wedge m_{i}\neq bg$ **do**7:        **for** $\left(j,m_{j}\right)\in N_{D}\left(i,m_{i}\right)\wedge m_{j}=bg\wedge j\notin V$ **do**8:           $push(r_{j},j,PQ)$9:           V←j∪V                        ▹ flag element as visited10:        **end for**11:    **end for**12:    **while** |PQ|≠0 **do**13:        i←pop(PQ)14:        $Nms\leftarrow \{m_{j}|m_{j}\in N_{D}\left(i,m_{i}\right)\wedge m_{j}\notin \left\{bg,wl\right\}\}$   ▹ neighbors markers of $\left(i,m_{i}\right)$15:        **if** |Nms|=1 **then**16:           $M\leftarrow \left(M\setminus \left(i,m_{i}\right)\right)\cup \left\{\left(i,m_{j}\right)|m_{j}\in Nms\right\}$17:           **for** $j\in \{k|\left(k,m_{k}\right)\in N_{D}\left(i,m_{i}\right)\wedge k\notin V\}$ **do**18:               $push(max\left(r_{i},r_{j}\right),j,PQ)$19:               V←j∪V20:           **end for**21:        **else**22:           $M\leftarrow \left(M\setminus \left(i,m_{i}\right)\right)\cup \left(i,wl\right)$                ▹ label element as WL23:        **end if**24:    **end while**25:    **return** *M*26:**end function**

The variables and steps of the algorithm by Meyer are the following:

M1 Image elements corresponding to markers are flagged as visited i∈V; see line 5 in Algorithm 2.
M2 Image elements having marked neighbors are added to the priority queue and flagged as visited; see lines 6–11.
M3 The element with the highest priority is extracted from the queue; if the priority queue is empty, then the algorithm terminates; see lines 12–13.
M4 If all marked neighbors Nms of the extracted element have the same marker, then the image element is labeled by that marker; if marked neighbors of the extracted element have different markers, then the elements are flagged as WL-belonged with marker wl; see lines 14–23.
M5 Nonflagged as visited neighbors of the extracted element are added to a priority queue (with same or lower priority) and flagged as visited if the extracted element is not WL-belonged; then, go to step M3; see lines 17–20.

**Figure 2:**
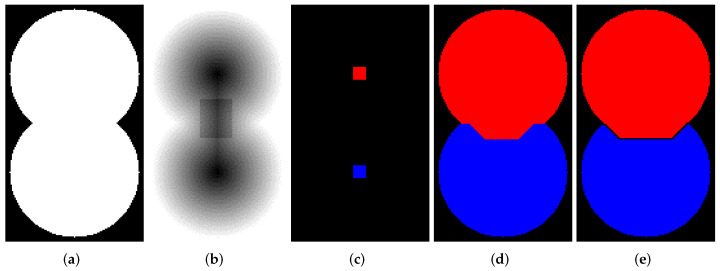
(**a**) Two overlapping binary discs; (**b**) relief; (**c**) initial markers; (**d**) segmentation result without watershed lines (WL) construction; (**e**) segmentation result with WL construction.

Let us consider how to operate the abovedescribed marked-controlled algorithms with WL [27] and without WL [25] construction. Figure 2a shows a binary image containing two overlapping discs. We generated this image by a simple code in Python. Then, we create a relief by subtracting a constant from a rectangular area in the center part of the inverted EDT result for image containing two overlapping disks (see Figure 2b). The image containing initial markers (red and blue) is generated by placing the red and blue squares in the centers of the discs (Figure 2c); correspondingly, the markers are located in local minima of the relief. Segmentation results obtained by algorithms with (Figure 2e) and without WL (Figure 2d) construction are different. This example refutes the common misconception that differences in outcomes of watershed with and without WL result only from image elements of WL. The example clearly shows that segmentation results can vary.

Over the 30-year history of the watershed, many algorithms have been developed: by topographic distance [28], via image foresting transform [29,30], rain falling [31,32], toboggan-based [33], via minimum spanning forest [34,35], hierarchical watersheds [36], etc. Surveys [37,38,39] compare various approaches for a watershed calculation. However, despite the enormous efforts to formulate the various mathematical concepts of watershed, only flooding-based algorithms are implemented in well-known software libraries [19].

Although all flooding-based implementations have estimation of computational complexity as O(N), where N is the number of image elements, its processing speed strongly depends on the used data structures, applied programming language, software optimizations, asymptotic constant, and other parameters [40]. Hendriks [41] performed research on various priority queues in terms of performance and demonstrated the importance of selecting the appropriate priority queue realization. For digital elevation models (DEM) used in geographic information systems (GIS), Barnes et al. [42] showed how the choice of different queues affects a performance of flooding-based watershed algorithms.

## 3. Implementations under Analysis

To select items for our analysis, we looked for the open-source and proprietary software that are used in practice for watershed segmentation of 3D images. For this search, we used Google^®^ and GitHub^®^ search engines, but also an IEEE Xplore database and the Google Scholar engine for papers that describe the practically used libraries for watershed segmentation, as well as the implementations of their algorithms. Table 1 contains information about the version of software used in our benchmarking, the license, the programming language applied in development of corresponding software, and the name of the function under analysis. Functions for marker-controlled watershed were selected except MATLAB^®^ and Octave that have only the conventional watershed, which starts flooding from local minima. For a fair comparison of MATLAB^®^ and Octave, we used relief images with the same locations of local minima as markers for other marker-controlled implementations.

We deal with the watershed segmentation implemented in the following seven open-source software libraries: Insight Segmentation and Registration Toolkit (ITK) (https://itk.org/ (accessed on 14 March 2022)) [43,44], Mahotas (https://github.com/luispedro/mahotas (accessed on 14 March 2022)) [45], Mathematical Morphology Image Library (Mamba) (http://mamba-image.org/ (accessed on 14 March 2022)) [46], Scikit-image (Skimage) (https://scikit-image.org/ (accessed on 14 March 2022)) [47], Simple Morphological Image Library (SMIL) (https://github.com/MinesParis-MorphoMath/smil (accessed on 14 March 2022)) [48], Octave (https://www.gnu.org/software/octave/ (accessed on 14 March 2022)) [49], and plug-in MorphoLibJ (https://imagej.net/plugins/morpholibj (accessed on 14 March 2022)) [50] for ImageJ [51] (below we refer to this realization as ImageJ). We also analyzed watershed implementations in three commercial products: a powerful set of tools for scientific and engineering calculations MATLAB^®^ by MathWorks (https://www.mathworks.com (accessed on 14 March 2022)), software for CT and Microscopy image data visualization and analysis Avizo^®^ by ThermoFisher Scientific (https://www.thermofisher.com (accessed on 14 March 2022)), and optimized software library for 2D and 3D image processing IPSDK by Reactiv’IP SAS (https://www.reactivip.com (accessed on 14 March 2022)). Indeed, there may be additional proprietary software for watershed segmentation applications, but we selected these three products. We are confident that a skilled researcher can conduct performance testing of other implementations for their purposes using our methodology.

Table 2 contains the following features of watershed implementations for volumetric image processing: available data types for relief image, connectivity, whether there are versions of the algorithm with and without WL construction, whether a mask can be used inside an algorithm, or whether or not there is a parallel code.

**Figure 3:**
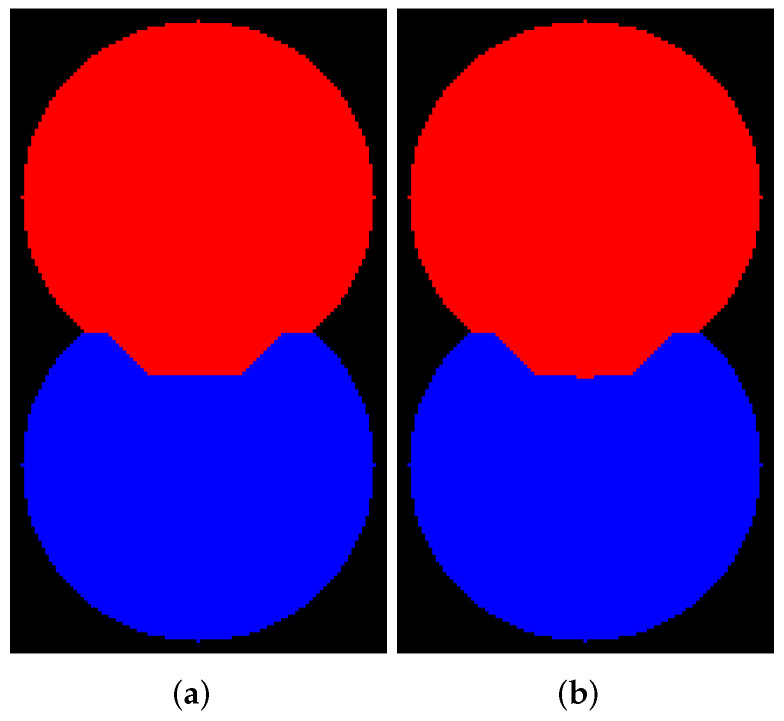
Segmentation outcome for relief from Figure 2b: (**a**) float32 data, (**b**) int16 data.

The quantization level of a relief image can affect the segmentation results. Figure 3 demonstrates two outcomes of segmentation by the Beucher and Meyer algorithm [25] for relief depicted in Figure 2b that was calculated as float32 and int16 data accordingly. Of course, a difference in this example is not big. However, it is worth noting that storing elements of a relief as floating-point or long integer enable us to obtain more accurate results, but it leads to greater memory consumption.

Scikit-image can handle arbitrary floating point and integer NumPy array as an input relief, but internally the array is cast to double the floating point type before watershed. Because we handle ITK via Python, we indicate in the table input the data types available from Python. ITK library in C++ has a wider possibility in sense data types. SMIL contains source code for creating images with float and double float types. However, at the moment, when compiling these types, compilation errors occur. The implementation of the watershed algorithm itself is not intended to process these types, since the priority queue size is calculated from the range of possible values for the image data type. For floating point numbers, this range is not fixed.

**Figure 4:**
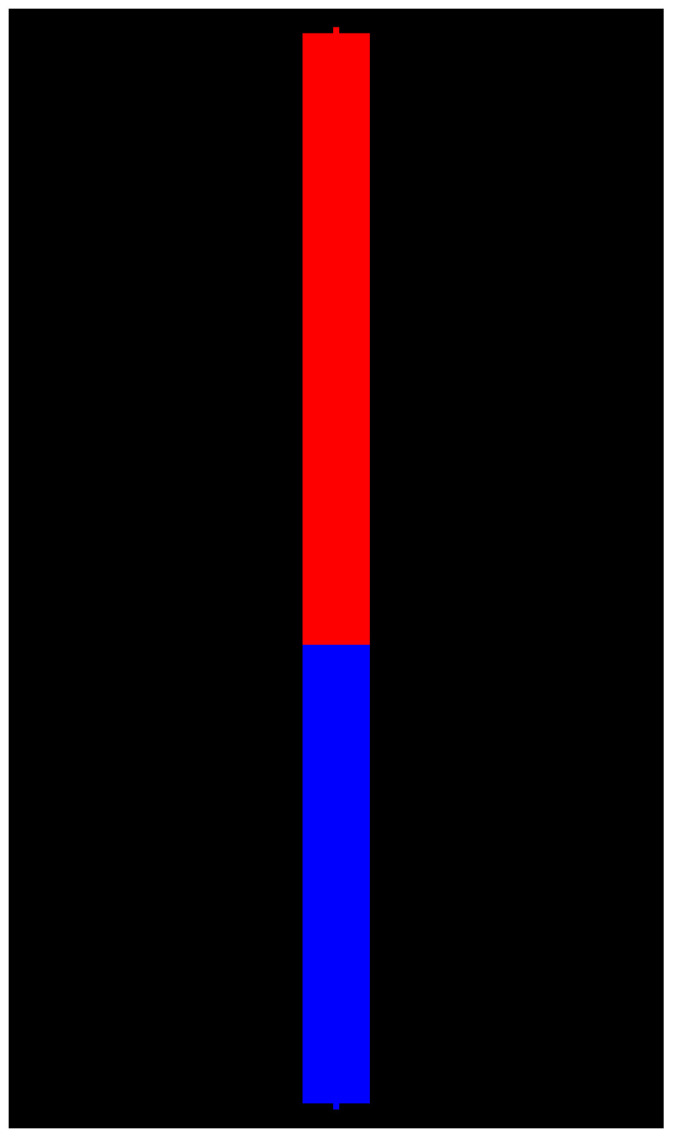
Result of watershed segmentation for relief from Figure 2b and markers from Figure 2c with 2-connectivity defined by vertical structural element.

IPSDK, Mamba, and probably Avizo^®^ (although we do not have exact information) enable us to use 26-connectivity only. ImageJ and ITK are able to apply 6- or 26-connectivity. For others, an arbitrary connectivity can be set by a 3 × 3 × 3 matrix. Surely, the use of custom connectivity is exotic, but perhaps it can be valuable in some applications. Figure 4 illustrates the segmentation result for relief from Figure 2b and markers from Figure 2c for a vertical structural element $\left[\begin{array}{ccc} 0 & 1 & 0\\ 0 & 1 & 0\\ 0 & 1 & 0 \end{array}\right]$.

All analyzed software except Mahotas have an algorithm with WL construction. Mahotas has a modification of the Mayers’s algorithm without WL, and it can return WL in a separate array, but produced watershed lines are frequently improper. Avizo, IPSDK, MATLAB, and Octave have no implementation of watershed without WL, other software have such a feature. We viewed codes of all open-source software under consideration and noticed that watershed implementations are based on algorithm by Beucher and Meyer (without WL) [25] and Meyers’s method [27] (with WL) or one from their modifications enumerated in [42].

**Figure 5:**
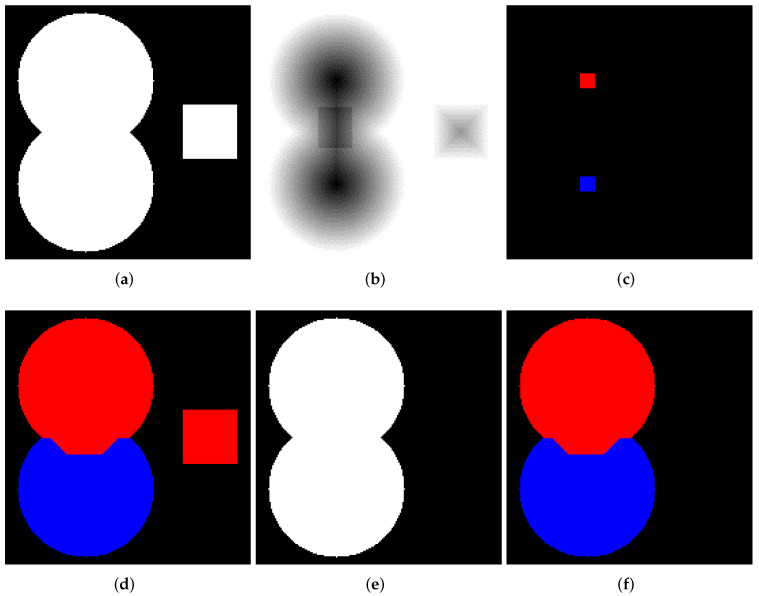
(**a**) Two overlapping discs and a square; (**b**) a relief based on modified result of Euclidean distance transform of image from (**a**); (**c**) initial markers; (**d**) result of the watershed without mask considering; (**e**) mask; (**f**) result of the watershed operated only inside given mask.

The use of a masked watershed can be valuable for many tasks. In addition, a calculation of watershed inside masked elements only allows us to reduce processing time. Images in Figure 5 serve to illustrate masked watershed operation. Figure 5a shows a binary image containing two overlapping discs and squares. The image was generated by code in Python. Then, we create a relief by subtracting a constant from a rectangular area inside the discs of the inverted EDT result for image from Figure 5a. Figure 5b shows the relief. The markers image is generated by placing red and blue squares in the centers of the discs (see Figure 5c). Figure 5d demonstrates the outcome of segmentation by watershed without WL construction for relief from Figure 5b. The mask in Figure 5e is a binary image containing only two overlapping disks from Figure 5a. Figure 5f demonstrates the outcome of the masked watershed without WL. These two segmentation results are different. We emphasize that in the general case, using a mask in a watershed algorithm is not equivalent to applying a mask to a watershed outcome or an input relief. Only Avizo, ImageJ, and Scikit-image have implementation of a masked watershed.

A concurrent execution is able to speed up watershed significantly. Unfortunately, only commercial software Avizo and IPSDK have parallel implementations of watershed. Moreover, both Avizo and IPSDK have two versions of watershed: repeatable and fast (or optimized speed). An outcome of the repeatable version is the same as by the consecutive watershed. Each execution of the fast parallel version provides a slightly different result. We assume that fast versions sacrifice strict synchronization between threads during priority queue processing to achieve a high processing speed.

## 4. Results

### 4.1. Measurement Procedure

We estimated the execution time and memory consumption of the various watershed implementations on a workstation with 2 CPUs Intel^®^ Xeon E5-2630 v3 (32 logical cores) @ 2.4 GHz and 128 Gb of RAM. We used 64-bit Microsoft^®^ Windows^®^ 10 Enterprise operating system (OS). When using other hardware and OS, the result may be different, but the ratio of the results in most cases should be preserved.

Corresponding watershed functions from IPSDK, ITK, Mahotas, Mamba, scikit-image, and SMIL are called via Python 3.8.8. Mamba and SMIL libraries were compiled by Microsoft^®^ Visual C++ 14.16 compiler. We applied the function *perf_counter* from the *time* module for processing time estimation. For the estimation of peak memory usage, we used the Python *memory-profiler* module (https://pypi.org/project/memory-profiler (accessed on 14 March 2022)).

For time measurements of watershed in other software under consideration, we employ: *tic* and *toc* functions for MATLAB and Octave; Python *time* module in Avizo^®^. ImageJ plug-in reports the processing time itself. For estimation of peak memory consumption in MATLAB^®^, Octave, and ImageJ, we used the Process Explorer software tool (https://docs.microsoft.com/en-us/sysinternals/downloads/process-explorer (accessed on 14 March 2022)). We did not measure memory consumption for Avizo^®^.

Processing time measurements were carried out several times and averaged:

(1) $$T=\frac{1}{K}\sum_{i=0}^{K-1}\left(T1_{i}-T0_{i}\right),$$

where *K* is the number of measurements, K=10; $T1_{i}$ is the time after calling the watershed function; $T0_{i}$ is the time before calling the watershed.

A similar formula for estimation of memory consumption was used. Source codes of our benchmarking can be found at https://github.com/ant-Korn/Comparing_watersheds (accessed on 14 March 2022). For processing 3D images, only 26-connectivity was considered. We used a relief image as uint16 data for all software except Mamba and Scikit-image, where uint32 and float64 are used correspondingly.

### 4.2. Implementations with and without WL Construction

**Figure 6:**
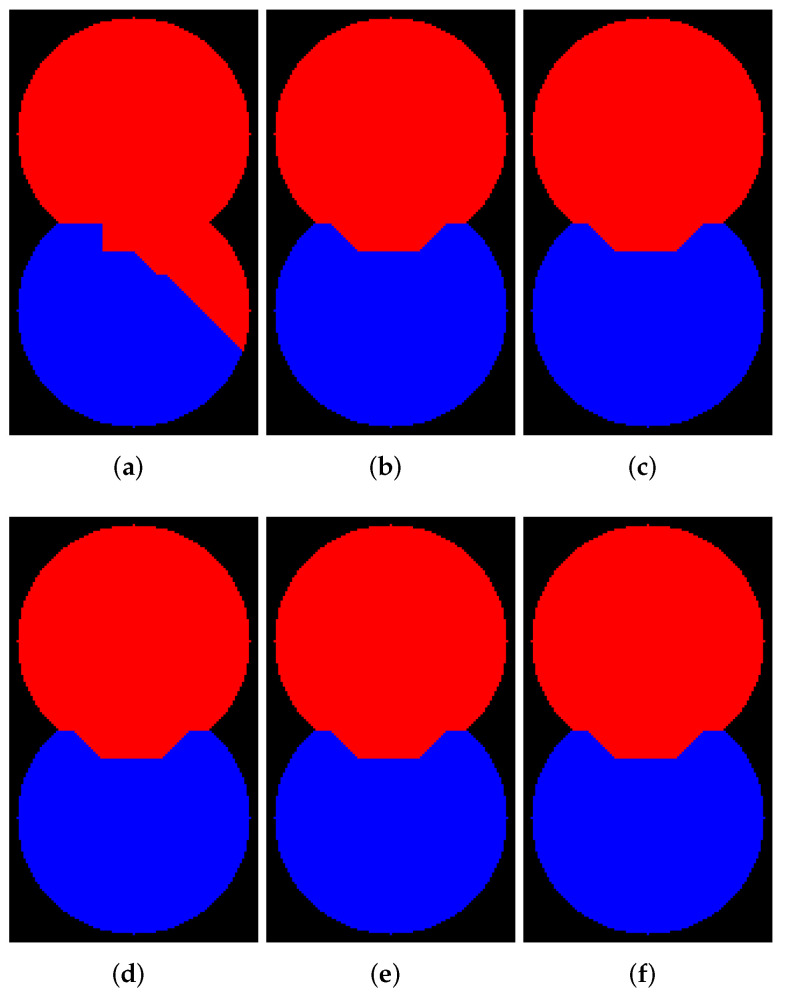
Results of segmentation via watershed without WL (8-connectivity): (**a**) ImageJ; (**b**) ITK; (**c**) Mahotas; (**d**) Mamba; (**e**) Scikit-image; (**f**) SMIL.

**Figure 7:**
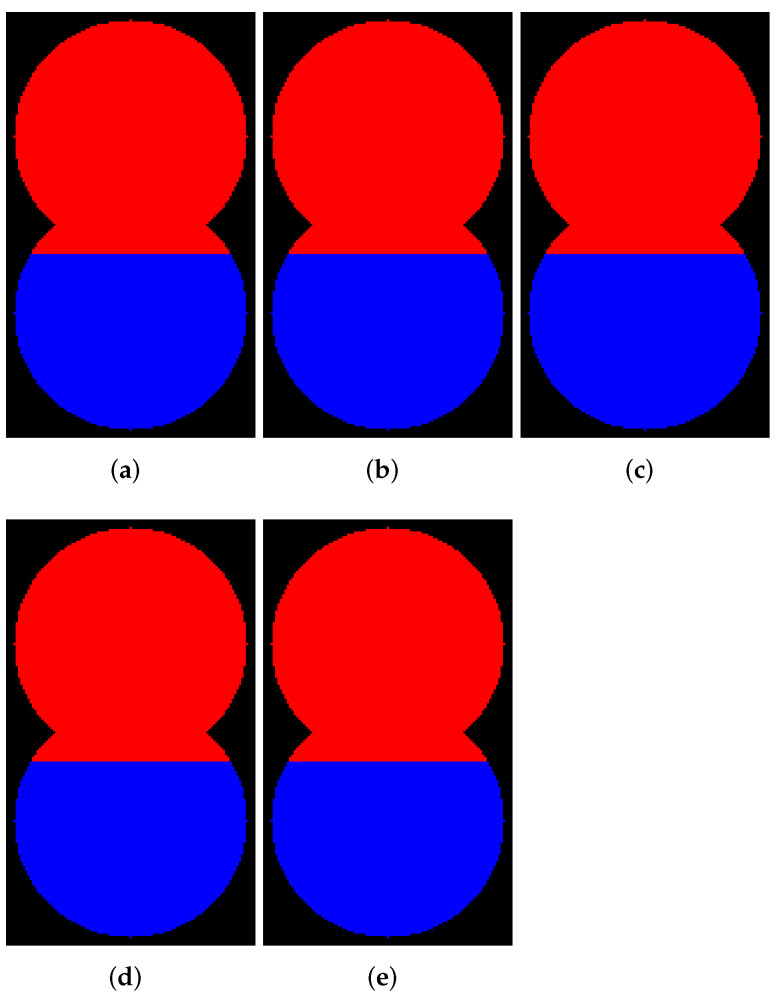
Results of segmentation via watershed without WL (4-connectivity): (**a**) ImageJ; (**b**) ITK; (**c**) Mahotas; (**d**) Scikit-image; (**e**) SMIL.

**Figure 8:**
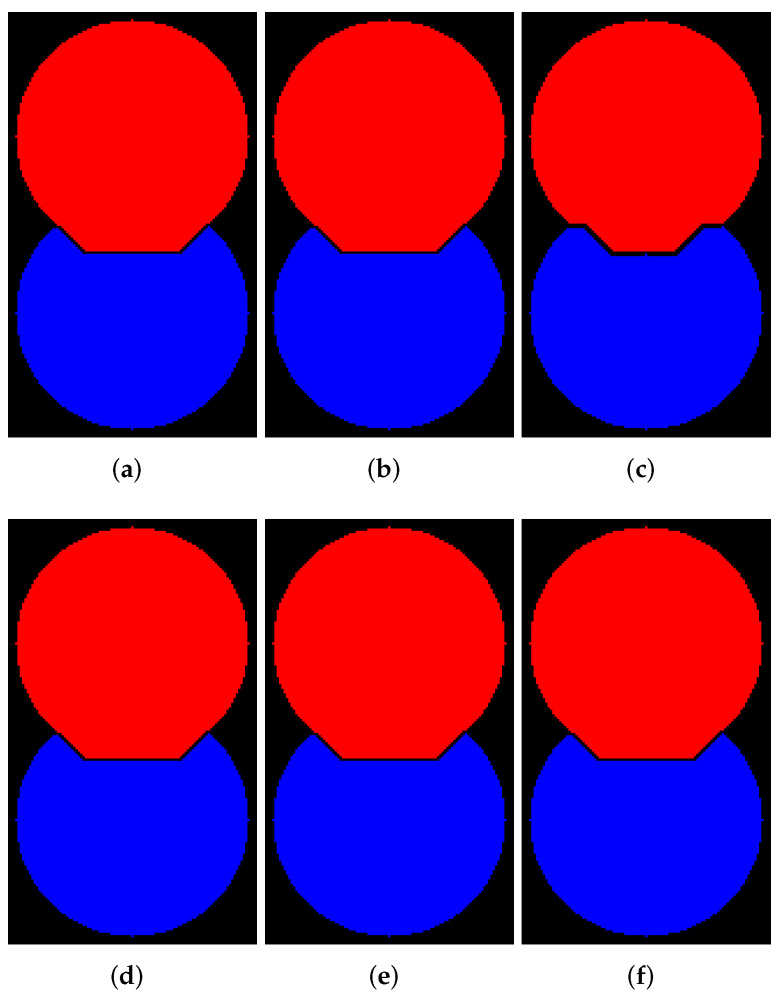
Results of segmentation via watershed with WL (8-connectivity): (**a**) ImageJ; (**b**) ITK; (**c**) Mahotas; (**d**) Mamba; (**e**) Scikit-image; (**f**) SMIL.

**Figure 9:**
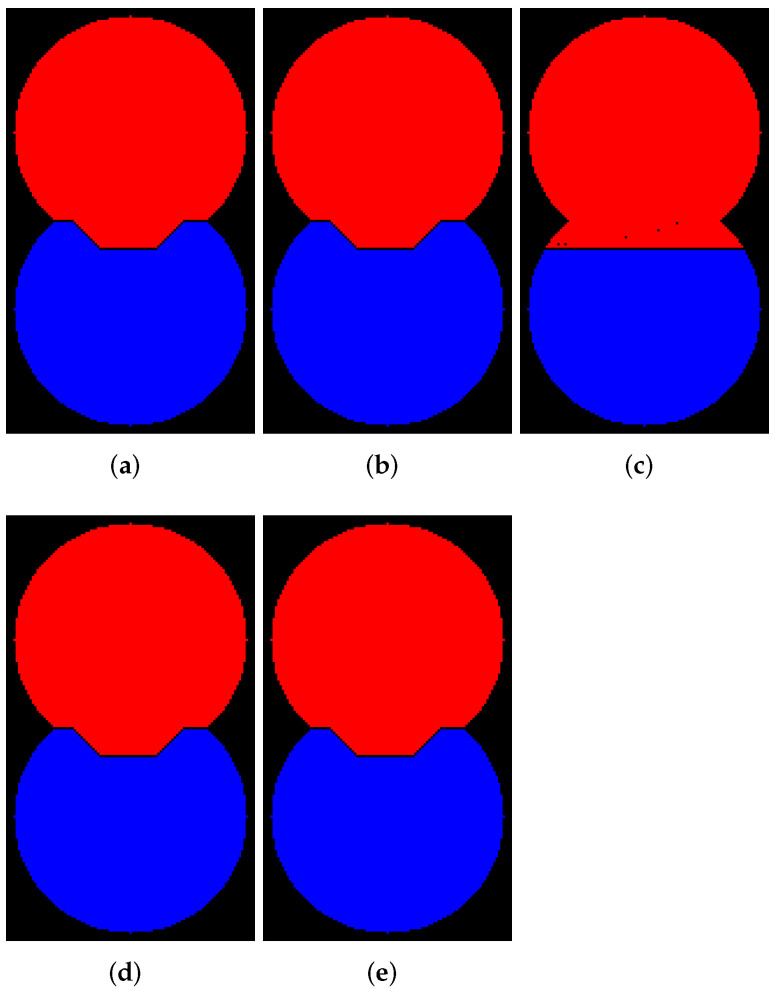
Results of segmentation via watershed with WL (4-connectivity): (**a**) ImageJ; (**b**) ITK; (**c**) Mahotas; (**d**) Scikit-image; (**e**) SMIL.

In this subsection, we demonstrate examples of marker-controlled watershed by those software that have the option to create results with and without WL. Figure 6, Figure 7, Figure 8 and Figure 9 contain segmentation outcomes for image and markers from Figure 2b,c processed by ITK, Mahotas, Mamba, SMIL, Scikit-image, and ImageJ for different types of connectivity. The aims are to once again demonstrate the differences for algorithms with and without WL construction as well as investigate peculiarities of various software implementations of watershed.

Figure 6 and Figure 7 visualize the results of segmentation via watershed without WL for 8- and 4-connectivity, respectively. Mamba supports 8-connectivity only. Certainly, the results of algorithms for 4- and 8-connectivity differ. In our opinion, outcomes of watershed with 8-connectivity look more reasonable for that relief rather than 4-connectivity, except for ImageJ, which produces an incorrect result for 8-connectivity.

Figure 8 and Figure 9 visualize the results of segmentation via watershed with WL construction for 8- and 4-connectivity, respectively. Mahotas produces results that differ from others; moreover, an outcome for 4-connectivity is improper. It is worth noting that the result of the algorithm with WL construction and 4-connectivity looks very similar to the outcome of the algorithm without WL construction and 8-connectivity.

In addition to illustrations, it is preferable to characterize a difference between various implementation by quantitative measures. Segmentation quality metrics can serve for the purpose. An accuracy for the results computed only for pixels inside the binary mask from Figure 5a is calculated by the following statement:

(2) $$Accuracy\left(\hat{Y},Y,M\right)=\frac{1}{\sum_{\begin{array}{c} i=0\\ m_{i}\neq 0 \end{array}}^{N-1}m_{i}}\sum_{\begin{array}{c} i=0\\ m_{i}\neq 0 \end{array}}^{N-1}1\left({\hat{y}}_{i}=y_{i}\right),$$

where *N* is the number of image pixels; $m_{i}\in M$ are the pixels of binary mask; ${\hat{y}}_{i}\in \hat{Y}$ are pixels of an image, for which we evaluate the quality of segmentation; $y_{i}\in Y$ are pixels of a ground truth image; 1(x) is the indicator function [52].

Naturally, we have no ground truth image in our benchmarking. However, segmentation outcomes by any implementation can be considered as ground truth to compare different realization with each other. As the ground truth images we define outcomes of SMIL. Table 3 contains accuracy for the segmentation results shown in Figure 6, Figure 7, Figure 8 and Figure 9.

### 4.3. Processing Time and Consumed Memory vs. 3D Image Size

**Figure 10:**
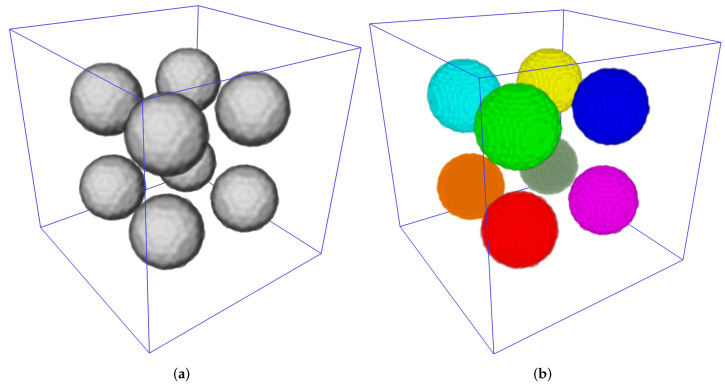
(**a**) 3D image containing balls. (**b**) Segmentation result.

To estimate processing time and memory consumed depending on image size, we developed a procedure for generation of a 3D synthetic binary image containing eight untouching balls. Surely, a simpler connected component labeling algorithm could be used for segmentation of such an image, but it is applicable for benchmarking of various watershed implementations as well. We placed eight initial unique markers in the centers of balls. Figure 10 demonstrates a volumetric image containing balls and instances segmentation result via a marker-controlled watershed.

Because the algorithm with and without WL construction differ, we measured the processing time and memory consumption separately for both types of watershed. For software products having two versions for the concurrent execution: repeatable and fast, we handled them with both versions.

**Figure 11:**
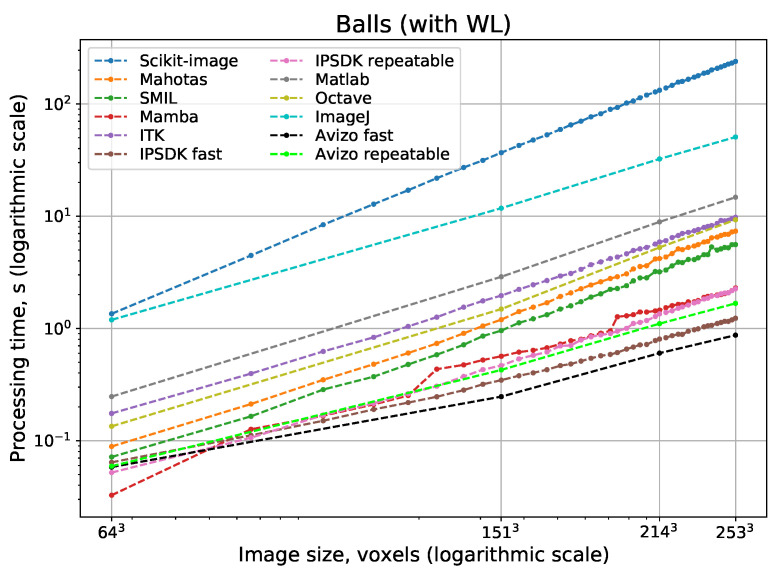
Processing time for watershed with WL depending on 3D image size.

**Figure 12:**
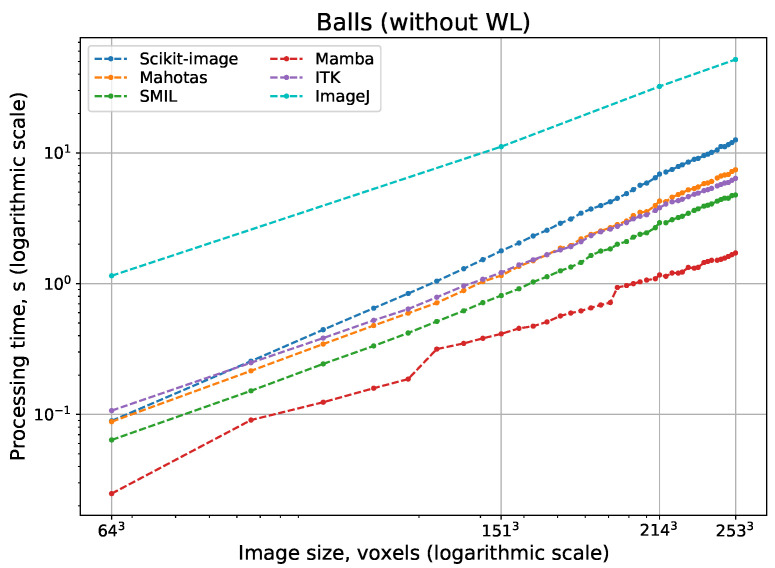
Processing time for watershed without WL depending on 3D image size.

Figure 11 and Figure 12 show logarithmic scale plots of processing time vs. image size for watershed implementations with and without WL, respectively. Indeed, a computational complexity for all implementations is O(N), but processing speed depend on implementation peculiarities significantly. The difference between the fastest and slowest realizations of watershed can be some orders of magnitude. Surprisingly, ImageJ and Scikit-image have the slowest watershed realizations, although these open-source packages are the most popular. Expectedly, fast parallel implementations in Avizo and IPSDK demonstrate the highest processing speed. Their parallel versions providing repeatable output perform better than any other solutions that use consecutive processing. Mamba and SMIL have a shorter processing time in comparison with other open-source software. For software that has the option to do watershed with or without WL, processing with the construction of WL requires more time than without it.

A high memory requirement can be a bottleneck to use watershed for processing of huge volumetric images. A good example of such 3D images is provided by X-ray micro-CT of sizes 1024 × 1024 × 1000, 2048 × 2048 × 1000, and 4000 × 4000 × 2000, which are used for the analysis of mineral particles [5,53,54]. To the best of our knowledge, there is no theoretical estimation of required memory for watershed algorithms in the literature. We are trying to do an estimation. Watershed algorithms take arrays of relief, markers and, optionally, a mask as input parameters, allocate space for priority queue, and create an array with segmentation result. Thus, theoretically, memory consumption grows linearly with the number of image elements. However, in practice, it strongly depends on data types of the arrays and queue. Additional overhead caused by the programming language takes place as well.

**Figure 13:**
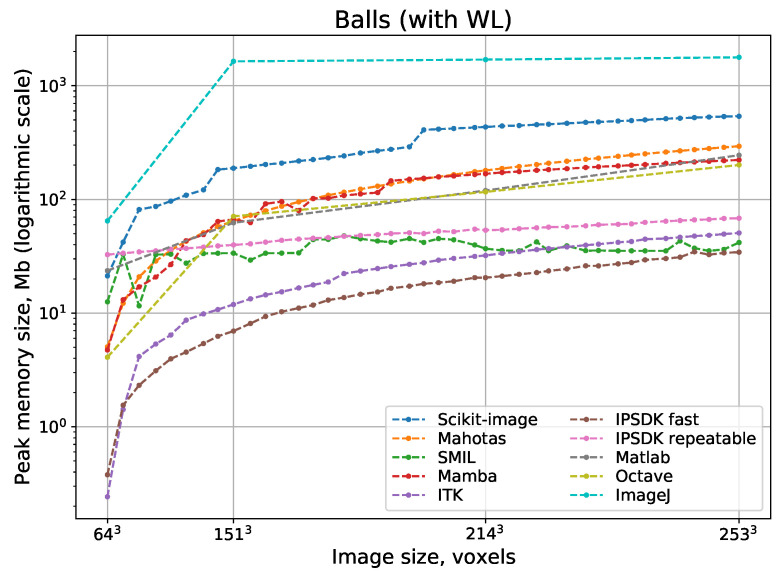
Peak memory consumption for watershed with WL depending on 3D image size.

**Figure 14:**
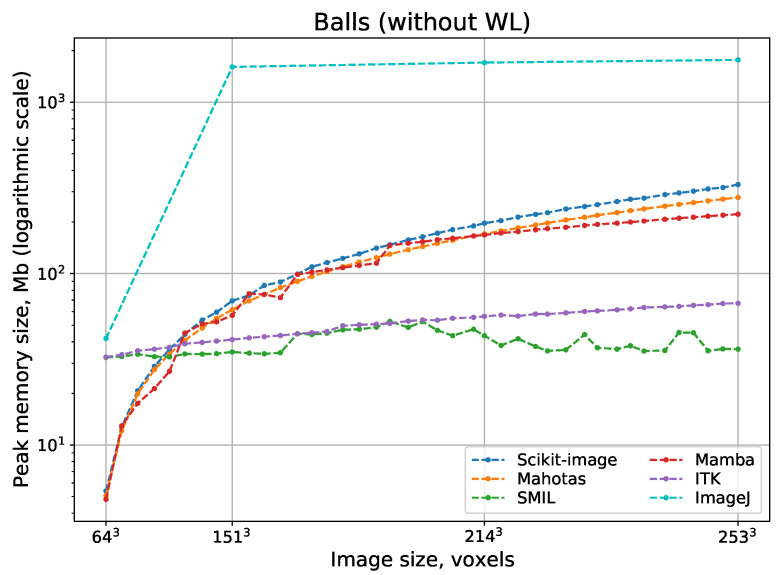
Peak memory consumption for watershed without WL depending on 3D image size.

Figure 13 and Figure 14, which show logarithmic scale plots of peak memory consumption vs. image size for watershed implementations with and without WL, support our theoretical assumptions. Similar to processing time, the difference in peak memory usage between the best and the worst realizations of watershed can be some orders of magnitude. ImageJ and Scikit-image consume the largest volume of memory. Proprietary IPSDK, as well as open-source SMIL and ITK require the smallest memory size. For software that has the option to conduct watershed with or without WL, peak memory consumption can be approximately the same (for example, SMIL and Mamba), or allocated memory volume for the version with the construction of WL is notably bigger (for example, Scikit-image).

### 4.4. Semantic Segmentation of FIB-SEM Image

In this subsection, we consider a real-world problem, namely semantic segmentation of a volumetric image obtained by focused ion beam scanning electron microscope (FIB-SEM). The aim of segmentation is to generate a digital twin of the rock sample for further mathematical simulations used in digital rock physics [55]. In the segmentation procedure, a voxel is classified as relating to solid or pore. One of the main specific features of FIB-SEM images of porous media is referred to as the pore-back or shine- through effect. Because pores are transparent, their back side is visible in the current slice whereas, in fact, it actually lies in the next slices [56].

**Figure 15:**
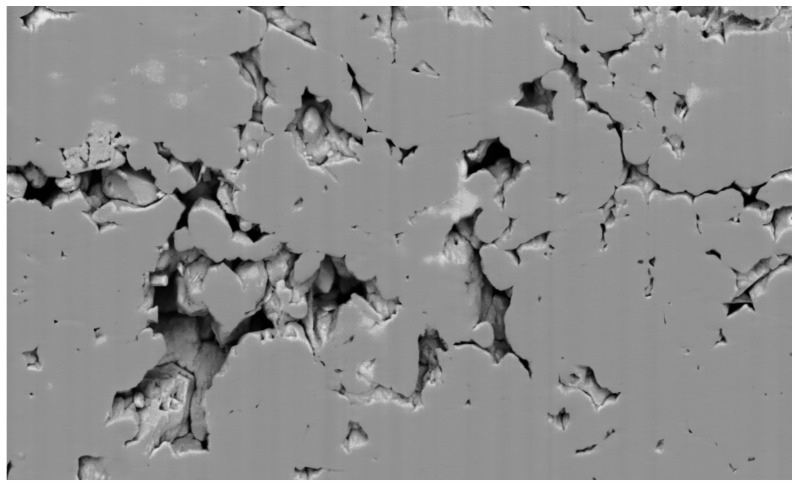
A slice of an FIB-SEM image of a sample of carbonate rock.

Figure 15 shows a 2D slice of a 3D FIB-SEM image of a sample of carbonate rock. The stack of slices was acquired by FEI Helios NanoLab 660 DualBeam™ system. The 3D image composed from these slices has a size of 1394 × 841 × 929 voxels; i.e., the number of image elements is about $10^{9}$.

Because pore-back effect segmentation of FIB-SEM images is a challenging problem, several approaches based on marker-controlled watershed were proposed to solve it [57,58]. These approaches include those where relief is a combination of the pre-processed FIB-SEM image and the results of its morphological processing, and markers of pores and solid are the result of thresholding of various filter outputs. In considering cases, all voxels of images should be classified; accordingly, a mask application to limit the number of processed voxels is unusable. Voxels of the initial markers can occupy a significant part of an image; the typical percentage of initially marked voxels is from 30 to 50%.

Table 4 contains the processing time and peak memory size of various watershed implementations for the segmentation of an FIB-SEM image. Supposedly, a construction of WL is unnecessary for the given problem. Nevertheless, we evaluate consumed resources for both algorithms with and without WL construction. For proprietary software having two versions for parallel execution, repeatable and fast, we handle both of them. Regarding MATLAB and Octave, which have no function for the marker-controlled watershed, in contrast to other tasks considered in the previous and next subsections, we were unable to obtain a proper outcome, so their outcomes in Table 4 can be considered rough estimates only.

Parallel realizations in commercial Avizo and IPSDK provide the fastest processing speed, which is about 0.5 min. From open-source software libraries, Mamba has the highest result, at about 3 min. SMIL and ITK process a given FIB-SEM image in about 10 min. Watershed execution time of an Scikit-image with WL construction is absolutely unacceptable. The version of the Scikit-image without WL construction works in about 40 min, but its watershed with WL operates 6.5 times longer. In general, the difference between the worst processing time (4.5 h) and the best time (0.5 min), is colossal depending on the used software implementation.

The differences in peak memory usage between various watershed realizations is also considerable. IPSDK is the most memory efficient, using slightly more than 3 bytes per image element. We assume it uses uint16 for relief and uint8 for both initial and final markers. Other software libraries are more memory-wasteful. Mamba and ITK require 10 to 14 times more memory than the voxels number in an image. Peak memory size of Scikit-image and Mahotas is about 60 times more than the number of image elements. For example, to segment an image having only $1000^{3}$ voxels, it would require a workstation with at least 64 Gb memory.

**Figure 16:**
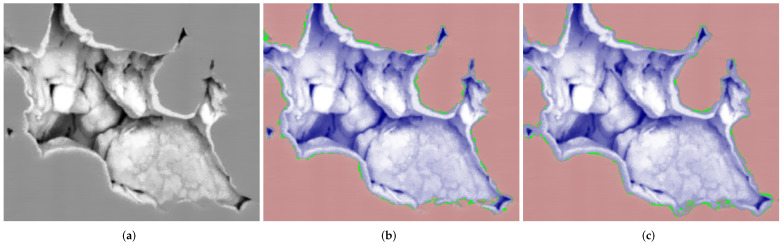
(**a**) Fragment of a slice from Figure 15; results of segmentation via (**b**) IPSDK and (**c**) Avizo: solid in red, pore space in blue, difference between repeatable and fast versions in green.

The fast versions of both parallel implementations operate several times faster in comparison with the repeatable ones. However, at first glance, it seems careless to use implementations that produce irreproducible results. At least, it is important to find the answers to the following questions: Is it suitable to use a realization that provides unrepeatable outcomes? Where is the place for voxels that alter for different program launches? How do those random changes affect the segmentation result? Figure 16 illustrates the results of segmentation using IPSDK and Avizo for fragments of a slice from Figure 15: voxels of a solid in red tones, voxels of pore space in blue, difference between repeatable and fast versions in green. Segmentation was conducted by the watershed-based method described in [58]. One can see, the altered image elements are near the border between classes. For both software, the percentage of changed voxels was about 1%. Changed voxels were distributed among the pores and solids approximately equally, i.e., the total amounts of voxels of both classes remained almost the same, but segmentation quality metrics such as accuracy and intersection-over-union (IoU) can vary several percent between results of fast version runs. Therefore, each researcher should decide depending on the application area, whether it is acceptable to obtain slightly irreproducible results by increasing the processing speed or not. For segmentation of a given FIB-SEM image, we prefer to apply the fast version of watershed due to the effect of changed voxels near the border being between classes, which is less than the uncertainty in the locations of such a border.

### 4.5. Instance Segmentation of 3D Binary Micro-CT Image

**Figure 17:**
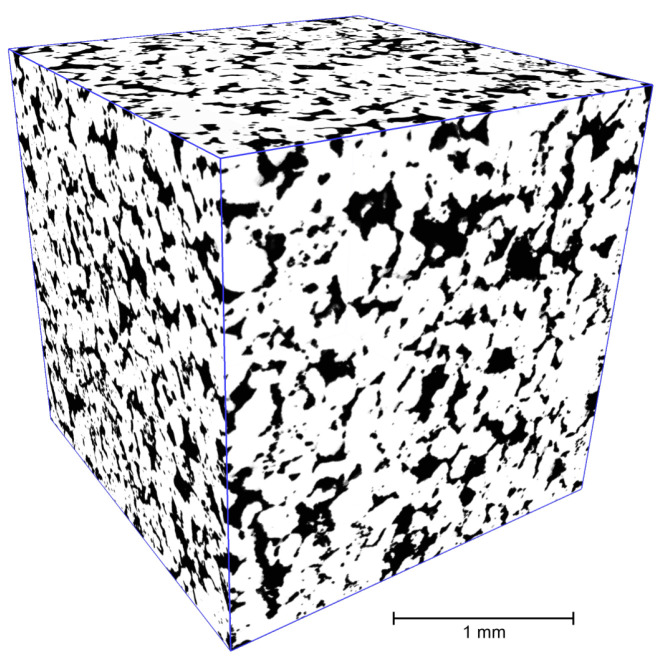
Digital twin of Buff Berea sandstone.

In this subsection, we consider the segmentation of a binary micro-CT image on pore bodies. The WL between pore bodies are pore throats [59]. An initial grayscale micro-CT image of Buff Berea sandstone was acquired by the Bruker Skyscan™ 1172 micro-CT system. Additional details about scanning parameters can be found in [60]. To produce a binary digital twin (see Figure 17), the *indicator kriging* algorithm [61,62] was used.

Then, we applied marker-controlled watershed to the inverted distance map (i.e., the result of 3D EDT) for this binary image. Markers are located in local minima of the inverted distance map. In contrast to the segmentation of the FIB-SEM image described in the previous subsection, initial markers occupy an insignificant part of the image volume. The size of the 3D image is $1000^{3}$ voxels. Porosity, that is, the percentage of voxels of pore space for which segmentation is performed, is about 30%.

The calculation of a distance map precedes the segmentation by watershed. It is interesting to assess the processing time of EDT implemented in various libraries. Besides the software mentioned in Section 3, Table 5 includes a performance measure of the corresponding EDT calculation functions for SciPy [63] and multilabel anisotropic 3D Euclidean distance transform (MLAEDT-3D) (https://github.com/seung-lab/euclidean-distance-transform-3d (accessed on 14 March 2022)), which applies an algorithm inspired by [64]. We did not carry out deep benchmarking, we estimated the processing time of the 3D distance transform for the image depicted in Figure 17. Similar to watershed, the difference in processing time of various EDT realizations is significant. IPSDK, MLAEDT-3D, and ITK achieve performance of about 10 s. Surprisingly, popular Avizo, ImageJ, and SciPy operate more than 10 times longer. Mahotas works unacceptably long.

Table 6 contains the processing time and peak memory consumption of various watershed implementations for segmentation of the distance map created for the binary micro-CT image. WL construction is required for a given problem because of the pore throat segmentation. Nevertheless, we evaluated the consumed resources for both algorithms with and without WL construction. In the considered task, only about one-third of voxels are processed, because we are interested in segmentation in pore space only. That is why using a mask, which indicates voxels for processing, is potentially able to speed up the segmentation. Therefore, we additionally evaluated resources for watershed realizations with masks for Scikit-image and ImageJ. Indeed, speeding up of processing takes place for implementations with the “mask” parameter. Again, we tested both fast and repeatable parallel versions of watershed in Avizo and IPSDK.

In general, the results in Table 4 and Table 6 coincide, but small differences appear. Because the numbers of image elements are approximately equal, it is essential to compare outcomes from both tables to each other. Similar to the previous case, parallel realizations in commercial Avizo and IPSDK provide the fastest processing speed; however, segmentation works several times longer vs. an operation with an FIB-SEM image. This is because, in a marker-controlled watershed of an FIB-SEM image, the initial markers occupy a notable part of the volume, leading to a smaller number of image elements that need to be labeled. Even though we are only interested in the segmentation of the pore space of a micro-CT image, watershed implementations operate on the entire image, except those capable of mask usage.

From open-source software libraries, SMIL, Mamba, and ITK process a given image from 11 to 20 min. Versions with WL construction operate longer from 10 to 30%. Again, watershed execution time of Scikit-image with WL construction is unacceptable, but Scikit-image without WL construction and with mask has a comparable performance to SMIL.

In general, for a given problem, total memory consumption is smaller than for FIB-SEM segmentation. Again, IPSDK is the most memory efficient. ITK uses about 5 bytes per image element, which is acceptable, as a rule. Memory consumption in SMIL is more or less acceptable, especially for the version without WL. Other software libraries require more memory. Again, peak memory size of Scikit-image is the largest.

**Figure 18:**
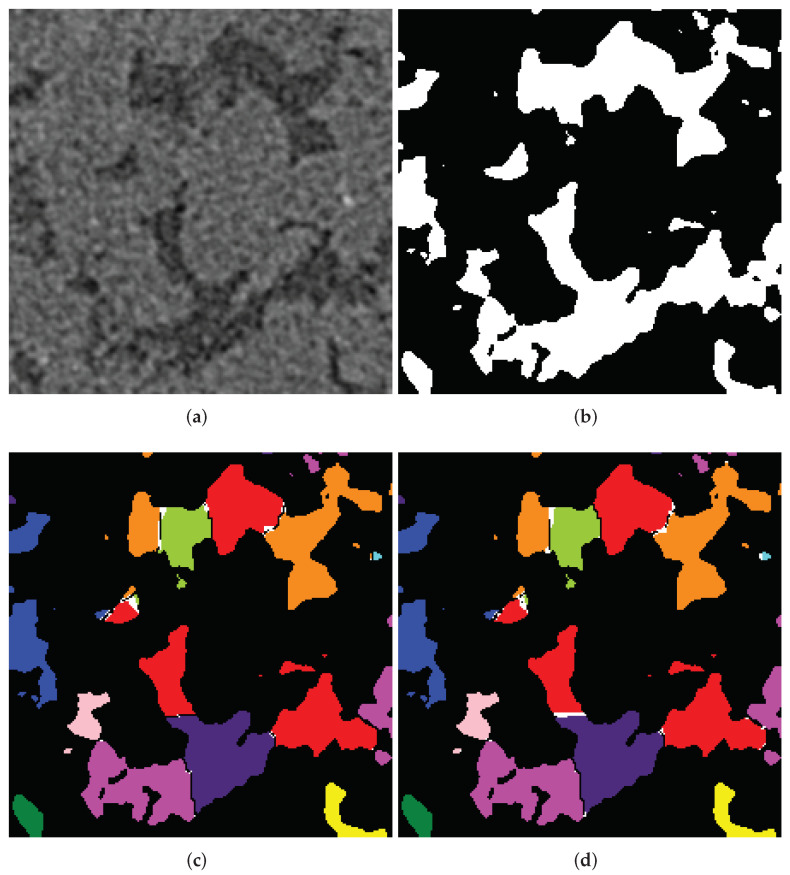
Fragment of (**a**) slice of the micro-CT image; (**b**) segmented pore space; results of segmentation via (**c**) IPSDK and (**d**) Avizo: pore bodies (different colors), difference between repeatable and fast versions (white).

Let us compare fast and repeatable versions of watershed for the segmentation of the distance map of a micro-CT image. Figure 18 shows a fragment of a grayscale slice of an initial micro-CT image of sandstone, the same fragment after semantic segmentation by indicator kriging and instance segmentation on pore bodies by fast versions of watershed in Avizo and IPSDK. Pore bodies are designated by various colors, and the difference between repeatable and fast versions is in white. Similar to the previous case, the altered image elements are near the border between classes. The percentage of changed voxels is about 1% for Avizo and 1.5% for IPSDK. Changed voxels are distributed among adjusted pore bodies approximately equally. Linear sizes, surface area, and volume of pore bodies vary insignificantly; however, measures of pore throats (dams between bodies) are more sensitive, especially for small throats. For segmentation of a given micro-CT image, we prefer to apply the repeatable version of watershed because the effect of changed voxels on pore throats in further simulations (e.g., modeling of capillary curves) can be notable.

## 5. Discussion

Segmentation of volumetric images by watershed has a high computational burden. In this paper, we have evaluated processing time and consumed memory of several marker-controlled watershed implementations. Despite the fact that most of them implement the same algorithms with O(N) time complexity relative to the number of image elements, our benchmarking shows differences in several orders of magnitude in execution time and used memory size, as it also depends on many other factors, such as implementation language, applied software optimizations, etc. In general, the commercial software under consideration demonstrates better performance in comparison with free publicly available ones. In regards to open-source libraries, SMIL and Mamba operate faster than others. However, you must be skilled in software engineering to apply those libraries. It is impossible to setup SMIL and Mamba by means of the well-known Python package managers *pip* and *conda*; however, they need to be built from C++ sources. We consider the best option of all the off-the-shelf open-source software packages to be ITK.

Undoubtedly, there is much room for improvement in watershed implementations. First, for some problems, simple tricks such as using masks to limit the number of processed voxels enable us to decrease execution time significantly. Second, to the best of our knowledge, existing watershed implementations are based on priority-flood algorithms [42], whereas other types of watersheds have many valuable findings that potentially improve performance or decrease the size of consumed memory. For example, in many publications, approaches for parallelization were proposed earlier [65,66,67,68,69,70,71,72,73]. Even several concepts for watershed realization in GPU (graphics processing unit) were depicted [74,75,76]. A concurrent execution is a promising way to speed up processing, but it is worth noting that parallel implementation of segmentation by watershed is not available in any of the open-source software considered in this study.

An insufficient memory size is a serious bottleneck dealing with huge 3D images. For handling such images, development of out-of-core algorithms [77], i.e., approaches that are capable of processing datasets larger than the main memory, are necessary. Open-source Python package PoreSpy (https://porespy.org/ (accessed on 14 March 2022)) [78] applies a prospective approach for processing of 3D images part-by-part. PoreSpy uses Scikit-image watershed implementation for overlapped chunks of initial data via Dask (https://dask.org (accessed on 14 March 2022)). The effectiveness of that technique depends on the set of parameters: the number of chunks, the number of overlapping voxels between them, the number of parallel processes for performing computations, etc. However, segmentation outcomes can differ near borders of the chunks in comparison with segmentation results for the entire image, and proper selection sizes of chunk and overlapping area is a nontrivial issue. Nevertheless, frequently in practice, such a divide-and-conquer approach is the only one possible. Moreover, a similar method can be applied for distributed computing of big volumetric images in the cloud. We confidently forecast a growth of investigation in the direction of distributed parallel processing of 3D images part-by-part in general and for segmentation by watershed in particular.

## Figures and Tables

**Table 1:** Watershed implementations under the review.

Software	Version	Language	Object/Function	License
Avizo 3D	2021.1	C++	Marker-Based Watershed	commercial
ImageJ	ImageJ 2.1.0/1.53c; MorphoLibJ 1.4.3.1	Java	legacy:inra.ijpb.plugins. MarkercontrolledWatershed3DPlugin	ImageJ BSD-2; MorphoLibJ LGPL-3.0
IPSDK	3.0.1.0	C++	seededWatershed3dImg	commercial
ITK	5.2.0	C++	MorphologicalWatershedFromMarkersImageFilter	Apache 2.0
Mahotas	1.4.11	C++	cwatershed	MIT
Mamba	2.0.2	C	basinSegment, watershedSegment	MIT
MATLAB	9.10.0 (R2021a)	C/C++	watershed	commercial
Octave	6.3.0; Package 2.12.0	C/C++	watershed	GPL
Scikit-image	0.18.0	Cython	segmentation.watershed	BSD-3
SMIL	0.11	C++	basins, watershed	BSD-style

**Table 2:** Features of the considered implementations of watershed for 3D images.

Software	Relief Data Type	Connectivity	Without WL	With WL	Masked	Parallel
Avizo 3D	int(8, 16, 32), uint(8, 16, 32), float(32, 64)	no info	-	+	+	+
ImageJ	float32, uint(8, 16)	6, 26	+	+	+	-
IPSDK	int(8, 16, 32), uint(8, 16, 32)	26	-	+	-	+
ITK	float(32, 64), int16, uint(8, 16)	6, 26	+	+	-	-
Mahotas	float(32, 64), int(8, 16, 32), uint(8, 16, 32)	custom	+	+	-	-
Mamba	uint(1, 8, 32)	26	+	+	-	-
MATLAB	float(32, 64), int(8, 16, 32, 64), uint(8, 16, 32, 64)	custom	-	+	-	-
Octave	float(32, 64), int(8, 16, 32, 64), uint(8, 16, 32, 64)	custom	-	+	-	-
Scikit-image	float64	custom	+	+	+	-
SMIL	int(8, 16, 32), uint(8, 16, 32, 64)	custom	+	+	-	-

+ means the feature is supported; - means the feature is not supported.

**Table 3:** Segmentation accuracy for various watershed implementations in comparison with SMIL.

Features	Accuracy				
	ImageJ	ITK	Mahotas	Mamba	Scikit-Image
Without WL, 8-connectivity	0.91	1	1	1	1
Without WL, 4-connectivity	1	1	1	-	1
With WL, 8-connectivity	1	1	0.98	1	1
With WL, 4-connectivity	1	1	0.96	-	1

- means that Mamba does not support 4-connectivity

**Table 4:** Processing time and peak memory consumption of various watershed implementations for segmentation of an FIB-SEM image.

WL	Software	Time, s	Peak Memory Usage, Gb
+	Avizo 3D (fast)	20.0	—
+	Avizo 3D (repeatable)	46.0	—
-	ImageJ	2258.5	20.54
+	ImageJ	2304.7	21.33
+	IPSDK (fast)	31.9	3.35
+	IPSDK (repeatable)	109.7	3.35
-	ITK	603.0	14.46
+	ITK	714.1	10.69
-	Mahotas	1512.1	57.34
+	Mahotas	1621.3	57.34
-	Mamba	165.7	12.18
+	Mamba	238.3	12.29
+	MATLAB	1104.7	21.40
+	Octave	848.3	34.83
-	Scikit-image	2491.2	58.32
+	Scikit-image	16,113.1	92.94
-	SMIL	598.6	21.79
+	SMIL	696.5	21.76

+ means version with WL construction; - means version without WL construction;—means that peak memory usage was not estimated for Avizo 3D.

**Table 5:** Processing time of 3D Euclidean distance transform implementations.

Software	Version	Object/Function	Time, s
Avizo 3D	2021.1	Distance Map	193.2
		Distance Map (signed)	829.8
ImageJ	2.1.0/1.53c	legacy:fiji.process3d.EDT	168.0
IPSDK	3.0.1.0	morpho.distanceMap3dImg	6.1
ITK	5.2.0	SignedMaurerDistanceMapImageFilter	15.4
Mahotas	1.4.11	distance	>1 day
MATLAB	9.10.0 (R2021a)	bwdist	64.3
MLAEDT-3D	2.1.2	edt	14.0
SciPy	1.5.3	ndimage.distance_transform_edt	589.7
SMIL	0.11	distanceEuclidean	83.6

**Table 6:** Processing time and peak memory consumption of various watershed implementations for segmentation of distance map of micro-CT image.

WL	Software	Time, s	Peak Memory Usage, Gb
+	Avizo 3D (fast)	109.2	—
+	Avizo 3D (repeatable)	319.8	—
-	ImageJ	4612.4	21.91
-	ImageJ (with “mask” parameter)	1004.9	10.45
+	ImageJ	4727.4	22.45
+	ImageJ (with “mask” parameter)	1036.8	11.54
+	IPSDK (fast)	67.2	3.65
+	IPSDK (repeatable)	316.4	2.80
-	ITK	770.7	5.04
+	ITK	1232.2	5.97
-	Mahotas	1428.4	19.44
+	Mahotas	1554.6	20.24
-	Mamba	801.82	11.45
+	Mamba	942.1	11.45
+	MATLAB	2235.6	25.3
+	Octave	1788.5	31.98
-	Scikit-image	3190.8	20.30
-	Scikit-image (with “mask” parameter)	684.4	19.63
+	Scikit-image	36,991.6	38.65
+	Scikit-image (with “mask” parameter)	8262.3	33.48
-	SMIL	673.3	6.71
+	SMIL	860.2	8.58

+ means version with WL construction; - means version without WL construction;—means that peak memory usage was not estimated for Avizo 3D.

## Data Availability

Python code for generation of synthetic images used in the benchmarking can be found at https://github.com/ant-Korn/Comparing_watersheds (accessed on 14 March 2022). Both natural volumetric images that support this study are available from the corresponding author upon reasonable request.

## Abbreviations

    The following abbreviations are used in this manuscript:

2D	two-dimensional
3D	three-dimensional
CT	computed tomography
DEM	digital elevation models
DNN	deep neural network
EDT	Euclidean distance transform
FIFO	first in first out
FIB-SEM	focused ion beam-scanning electron microscope
GIS	geographic information system
GPU	graphics processing unit
IoI	intersection-over-union
ITK	insight segmentation and registration toolkit
OS	operating system
SMIL	simple morphological image library
WL	watershed lines

## Appendix A

**Table:** **Listing A1.** An example implementation of the marker-controlled watershed algorithm without WL for 3D images and 26-connectivity in the Python programming language.

**from** queue **import** PriorityQueue
**import** numpy as np
**def** marker_controlled_watershed_3D_without_WL_26connectivity(relief, markers):
# *Step* *BM1:*
priority_queue = PriorityQueue()
it = np.nditer([relief, markers], flags=[’multi_index’])
**for** relief_value, marker_value **in** it:
**if** marker_value:
**for** ngb_z, ngb_y, ngb_x **in** neighbours_window(it.multi_index, markers):
**if not** markers[ngb_z, ngb_y, ngb_x]: *# not marked neighbor*
*# put coordinates of pixel with relief value priority:*
priority_queue.put((relief_value, it.multi_index))
**break**
**while not** priority_queue.empty():
*#* *Step* *BM2:*
current_priority, current_coordinates = priority_queue.get()
*#* *Step* *BM3:*
**for** ngb_z, ngb_y, ngb_x **in** neighbours_window(current_coordinates, markers):
**if** **not** markers[ngb_z, ngb_y, ngb_x]: *#* *not* *marked* *neighbor*
markers[ngb_z, ngb_y, ngb_x] = markers[current_coordinates]
*#* *Step* *BM4:*
relief_value = relief[ngb_z, ngb_y, ngb_x]
**if** relief_value <= current_priority:
priority_queue.put((current_priority, (ngb_z, ngb_y, ngb_x)))
**else:**
priority_queue.put((relief_value, (ngb_z, ngb_y, ngb_x)))
**return** markers
**def** neighbours_window(pixel_coordinates, markers):
z, y, x = pixel_coordinates
ngb_z_start, ngb_y_start, ngb_x_start = z - 1, y - 1, x - 1
ngb_z_stop, ngb_y_stop, ngb_x_stop = z + 2, y + 2, x + 2
offset_z, offset_y, offset_x = -1, -1, -1
*#* *checks* *of* *elements* *on* *the* *image* *border:*
**if** z == 0:
ngb_z_start, offset_z = 0, 0
**if** z == markers.shape[0] - 1:
ngb_z_stop = z + 1
**if** y == 0:
ngb_y_start, offset_y = 0, 0
**if** y == markers.shape[1] - 1:
ngb_y_stop = y + 1
**if** x == 0:
ngb_x_start, offset_x = 0, 0
**if** x == markers.shape[2] - 1:
ngb_x_stop = x + 1
*#* *neighbours* *window* *with* *current* *element:*
neighbours = markers[ngb_z_start:ngb_z_stop,
ngb_y_start:ngb_y_stop,
ngb_x_start:ngb_x_stop]
it = np.nditer([neighbours], flags=[’multi_index’], op_flags=[’readwrite’])
**for** marker_value **in** it:
ngb_z, ngb_y, ngb_x = it.multi_index
ngb_z, ngb_y, ngb_x = z + ngb_z + offset_z, \
y + ngb_y + offset_y, \
x + ngb_x + offset_x
yield ngb_z, ngb_y, ngb_x

**Table:** **Listing A2.** An example implementation of the marker-controlled watershed algorithm with WL for 3D images and 26-connectivity in the Python programming language.

**def** marker_controlled_watershed_3D_with_WL_26connectivity(relief, markers):
*# Step* *M1:*
visited = (markers != 0)
*# Step* *M2:*
priority_queue = PriorityQueue()
it = np.nditer([markers], flags=[’multi_index’])
** for **marker_value** in **it:
** if **marker_value:
** for **ngb_z, ngb_y, ngb_x** in **neighbours_window(it.multi_index, markers):
** if not** markers[ngb_z, ngb_y, ngb_x] **and** \
**not** visited[ngb_z, ngb_y, ngb_x]: *# not marked neighbor*
priority_queue.put((relief[ngb_z, ngb_y, ngb_x],
(ngb_z, ngb_y, ngb_x)))
visited[ngb_z, ngb_y, ngb_x] = True
WL_MARKER = markers.**max**() + 1
*# Step M3:*
**while** **not** priority_queue.empty():
current_priority, current_coordinates = priority_queue.get()
*# Step M4:*
nb_marker = None
** **for** **ngb_z, ngb_y, ngb_x** in **neighbours_window(current_coordinates markers):
** if **markers[ngb_z, ngb_y, ngb_x] **and** \
markers[ngb_z, ngb_y, ngb_x] != WL_MARKER:
** if **nb_marker** **is** **None:
nb_marker = markers[ngb_z, ngb_y, ngb_x]
**elif** nb_marker != markers[ngb_z, ngb_y, ngb_x]:
markers[current_coordinates] = WL_MARKER
**break**
** **if** **markers[current_coordinates] == 0:
markers[current_coordinates] = nb_marker
*# Step M5:*
** **for** **ngb_z, ngb_y, ngb_x** **in** **neighbours_window(current_coordinates, markers):
** if not** visited[ngb_z, ngb_y, ngb_x]:
relief_value = relief[ngb_z, ngb_y, ngb_x]
** if **relief_value <= current_priority:
priority_queue.put((current_priority, (ngb_z, ngb_y,
ngb_x)))
**else:**
priority_queue.put((relief_value, (ngb_z, ngb_y, ngb_x)))
visited[ngb_z, ngb_y, ngb_x] = True
**return** markers
